# Telerehabilitation for New Wheelchair Evaluations: A Retrospective Study of Patient Characteristics

**DOI:** 10.5195/ijt.2024.6630

**Published:** 2024-06-28

**Authors:** Chelsea McClammer, Elizabeth A. Choma, Richard M. Schein, Mark R. Schmeler, Gede Pramana, Jake Gliniak, Corey Morrow

**Affiliations:** 1 Department of Occupational Therapy, Whitworth University, Spokane, Washington, USA; 2 Department of Physical Therapy, Whitworth University, Spokane, Washington, USA; 3 Department of Rehabilitation Science and Technology, School of Health and Rehabilitation Sciences, University of Pittsburgh, Pittsburgh, Pennsylvania, USA; 4 Department of Occupational Therapy, College of Health Professions, Medical University of South Carolina, Charleston, South Carolina, USA

**Keywords:** Health services research, Telerehabilitation, Wheelchair evaluations

## Abstract

The purpose of this paper was to describe the clinical and personal factors of persons with disabilities (PwD) seeking a new wheelchair evaluation via telerehabilitation compared to in-person appointments. This retrospective cohort analysis used the Functional Mobility Assessment and Uniform Dataset, which is a nationwide registry with ongoing enrollment at 31 clinical sites of PwD seeking a new wheelchair evaluation. PwD were stratified into either a Telerehabilitation Group or In-Person Group. There were 1,669 PwD in the Telerehabilitation Group and 10,284 in the In-Person Group. The Telerehabilitation Group had a higher mean age and higher percentage of Progressively Acquired Disabilities than the In-Person Group. This project lays the groundwork for future comparative effectiveness studies, which may influence telerehabilitation reimbursement policies for wheelchair services.

There are over 15 million Americans with disabilities who use assistive mobility devices (AMDs) including manual and power wheelchairs, walkers, canes, and crutches, and evidence shows this number is increasing (Brault, 2012). Both walking aids (Bertrand et al., 2017) and wheeled devices (Salminen et al., 2009) have been shown to reduce fall risk, improve patient participation in activities, and improve quality of life by increasing independent mobility. However, the lack of appropriate training or improper fit of an AMD can lead to low user satisfaction and an increased risk of device abandonment (Howard et al., 2022; Scherer, 2017).

The proper prescription of an AMD is a complex process that involves various stakeholders including the device user, a prescribing physician, an occupational or physical therapist, and an assistive technology professional (ATP) (Betz et al., 2022). The Rehabilitation Engineering and Assistive Technology Society of North America (RESNA) identifies the provisioning process for wheelchair service delivery mode in their Wheelchair Service Provision Guide. The guide recommends that clients who require a wheelchair on a long-term basis should be referred to a therapist and supplier who are qualified and have experience in seating and mobility to provide the client with the most appropriate device (Arledge et al., 2011). Without proper fitting of a device, consumers risk being prescribed an inappropriate device that could negatively impact their health with use over time and result in unmet mobility needs. These negative effects may include unnecessary expenses, injury, and duplication of effort, all leading to the possibility of device abandonment and wasted resources (Arledge et al., 2011).

Although proper prescription of an AMD is important, improper fitting is sometimes unavoidable due to issues with healthcare accessibility (rural areas, transportation constraints, lack of family support), cost, or medical reasons (e.g., COVID-19 isolation) (Nuara et al., 2021). Uninsured individuals with chronic conditions, a common population with AMD needs, are more likely to experience transportation barriers and negative health consequences. In 2017, 5.8 million people in the United States delayed care because they did not have transportation (Cochran et al., 2022).

The COVID-19 pandemic fostered a rapid and strong push for the adoption of telerehabilitation technology and healthcare delivery research (Iodice et al., 2021). During the first week of March 2020, telehealth increased by about 400% and continued to rise throughout the pandemic. Telerehabilitation was particularly useful in limiting in-person interactions to limit the spread of COVID-19. Telerehabilitation may improve accessibility and can be done by phone or through videoconferencing (Hyder & Razzak, 2020), and saves the patient time by not having to commute to their appointment. Telerehabilitation is emerging as a viable option for the delivery of rehabilitation (Chen et al., 2020; Iodice et al., 2021) that may improve patient recovery outcomes, reduce economic burden, and minimize access disparity for rural and low-income patients.

Individuals in rural areas often have limited medical resources, lack access to specialized centers, and must travel long distances to seek medical treatment, restricting access to medical care for individuals in these areas. Furthermore, for individuals with sensation issues (e.g., spinal cord injury), prolonged sitting during travel can carry the potential risk of worsening a sore or decubitus ulcer (Sabharwal et al., 2001). This is also true for other mobility impairments where common barriers for healthcare include access to both the physical environment as well as to specialists (O'Day, Dautel, & Scheer, 2002). Telemedicine is being used to increase access and quality of healthcare for individuals with limited access to health services (Hatzakis et al., 2003). Potential benefits for persons with disabilities (PwD) using telerehabilitation for AMD evaluations include easier access to specialists for rural-dwelling individuals, easier appointment scheduling for both patients and clinicians and an enhanced ability to assess patient needs within their home environment (Ott et al., 2022).

Previous studies have suggested an overall high level of satisfaction with AMD evaluations via telerehabilitation (Ott et al., 2020) and similar effectiveness when compared to in-person evaluations (Schein et al., 2010; Bell et al., 2020). However, there is a lack of understanding regarding which individual characteristics are conducive to telerehabilitation use for device evaluation. Identifying personal and clinical factors associated with telerehabilitation use would lead to targeted research to reduce barriers and develop more personalized service delivery methods based on specific needs. Therefore, the objective of this study was to provide a descriptive analysis of demographic and clinical characteristics of PwD who have previously had an AMD evaluation via telerehabilitation.

## Methods

This study was a retrospective, cross-sectional cohort analysis of persons seeking an evaluation for a new mobility device via telerehabilitation. Participants were extracted from the Functional Mobility Assessment and Uniform Dataset Registry (FMA/UDS) (Schmeler et al., 2019). This study was deemed de-identified, nonhuman research, and exempt from Institutional Review Board oversight by the University of Pittsburgh.

### Dataset

The FMA/UDS is a nationwide registry of PwD with a referral for an AMD evaluation with occupational therapists, physical therapists, and ATPs contributing from over thirty clinics. The FMA/UDS was developed in part to allow for the large data analysis of AMD users to determine which devices and service delivery models are most effective in improving patient satisfaction with mobility and reducing secondary health risks like falls and pressure sores. Data collection is ongoing and began in 2019 just prior to the COVID-19 pandemic. The last participant in this cohort was recorded in December 2023. During the initial evaluation for a new AMD, rehabilitation suppliers based in the United States collaborate with clinicians to complete intake forms. The intake form includes a binary yes/no question that indicates if the evaluation occurred using telerehabilitation (Schmeler et al., 2019). All adult (18 years or older) PwD were extracted from the FMA/UDS for analysis in this study.

### Variables of Interest

Demographic variables of interest that could lead to more targeted telerehabilitation delivery for AMD evaluation from the FMA/UDS were age, gender, and insurance/payer type. Clinical factors used from the FMA/UDS were primary diagnosis, type of device used prior to evaluation (if any), and satisfaction with current functional mobility status.

#### Primary Diagnosis

Persons with disabilities have specific AMD needs that vary based on functional level and prognosis. There are 31 individual primary diagnoses in the FMA/UDS (Schmeler et al., 2019). Diagnoses are further sub-grouped by (1) congenital disability (i.e., cerebral palsy or spina bifida), (2) acutely acquired disability (i.e., spinal cord injury or stroke), or (3) progressively acquired disability (i.e., Parkinson's Disease or osteoarthritis). There may be certain primary diagnoses that require an in-person AMD evaluation for specialized equipment or needs.

#### Current Device Use

During the FMA/UDS evaluation, PwD report if they are currently using an AMD at baseline. AMD was categorized as canes/crutches/walkers, manual wheelchairs, power wheelchairs, power scooters, and transport wheelchairs. Manual wheelchairs were further categorized as either custom-fit manual wheelchairs or non-custom-fit manual wheelchairs. Power wheelchairs are categorized as Group 1, Group 2, and Group 3. Other devices included power scooters and transport chairs.

#### Satisfaction with Mobility

Additionally, PwD reported on their satisfaction with functional mobility as measured by the Functional Mobility Assessment (FMA) (Kumar et al., 2013). The FMA is an easy-to-administer patient-reported outcomes tool designed to measure the effectiveness of an AMD. The following categories are rated using a 7-point Likert ordinal scale: (1) carrying out daily routines, (2) comfort needs, (3) health needs, (4) operating independently, safely, and efficiently, (5) reaching and carrying out tasks at different surface heights, (6) transfers from one surface to another, (7) personal care, (8) indoor mobility, (9) outdoor mobility, and (10) personal and public transportation. The scores for each category are then aggregated into an overall continuous variable out of a possible sixty points. We reported the mean and standard deviation for each category to identify patient-perceived limitations in functional mobility.

### Statistical Analysis

Using descriptive statistics, continuous variables were described with means, medians, and standard deviations. Categorical variables were reported on with frequency counts and percentages. All results were stratified by a binary yes/no variable indicating the use of telerehabilitation for the new AMD evaluation. Participants were designated as either in the “Telerehabilitation Group” or the “In-Person Group” (Table 1). Persons with a missing response for the telerehabilitation variable were excluded from the study. T-tests and chi-squares were used to compare the two groups. All data were analyzed using SAS, version 9.4 (*SAS Institute, Inc.*).

**Table 1:** Demographic Characteristics

Characteristic	Telerehabilitation Group (n = 1,669)	In-Person Group (n = 10,284)	p-value
Mean Age in Years (SD)	71.9 (13.1)	61.4 (17.4)	<.001
Gender			0.98
Male	760 (44.7%)	4,677 (45.6%)	
Female	905 (54.4%)	5578 (54.4%)	
Race			.99
White	544 (65.9%)	4075 (63.8%)	
Black	252 (30.6%)	1905 (29.8%)	
Hispanic/Latino	21 (2.6%)	261 (4.1%)	
Asian	5 (0.6%)	101 (1.6%)	
Other	3 (0.4%)	43 (0.7%)	
Origin of Disability			<.001
Acutely Acquired n (%)	450 (27.1%)	2,793 (27.6%)	
Congenital n (%)	59 (3.6%)	1,448 (14.3%)	
Progressively Acquired n (%)	766 (46.2%)	3,528 (34.9%)	
Other/Unspecified	384 (23.2%)	2,342 (23.2%)	
Payer Type			<.001
Medicaid n (%)	17 (1.1%)	1,558 (15.8%)	
Medicaid Managed Care n (%)	12 (1.0%)	1,048 (10.7%)	
Medicare n (%)	346 (21.3%)	3,152 (32.1%)	
Medicare Managed Care n (%)	575 (35.5%)	1,932 (19.6%)	
Private Pay n (%)	32 (2.0%)	48 (0.5%)	
Private Insurance Fee for Service n (%)	108 (6.7%)	536 (5.5%)	
Private Insurance HMO n (%)	290 (17.9%)	1,012 (10.3%)	
Veteran's Administration n (%)	0 (0.0%)	8 (0.1%)	
Vocational Rehabilitation n (%)	1 (0.1%)	5 (0.1%)	
Worker's Compensation n (%)	5 (0.3%)	46 (0.5%)	
Other/Not Listed	236 (14.6%)	491 (5.0%)	
Mean Stature in centimeters (SD)	168.1 (12.4)	166.6 (13.5)	<.001
Mean Mass in kilograms (SD)	94.3 (32.5)	88.5 (33.5)	<.001

## Results

### Demographic Factors

There were 11,953 PwD in the entire cohort (Table 1). The mean age of the 1,669 participants in the Telerehabilitation group was 71.9 (SD 13.1) years, compared to the mean age of 10,284 participants in the In-Person group, which was 61.4 (SD 17.4) years. For the Telerehabilitation Group, the most common payer type was Medicare Managed Care (35.5%) followed by Medicare (21.3%), and then Private Insurance HMO (17.9%). For the In-Person Group, the three most common payer types were Medicare (32.1%), Medicare Managed Care (19.6%), and Medicaid (15.8%).

The most common diagnoses for the Telerehabilitation Group were in the sub-group of progressively acquired disability, at 46.2% of that population. The three most frequent diagnoses in the Telerehabilitation Group were Unspecified Neuromuscular disease (19.9%), osteoarthritis (16.2%), and stroke (13.4%). The most common diagnoses for the In-Person group were also in the Progressively Acquired Disability sub-group but represented only 34.9% of that population. The three most frequent diagnoses in the In-Person Group were also Unspecified Neuromuscular disease (10.6%), osteoarthritis (9.7%), and stroke (9.7%).

## Clinical Factors

The most common AMD that new patients arrived at their evaluation with (baseline) for the Telerehabilitation Group were Non-Custom Manual Wheelchair (28.2%), Cane/Crutches/Walker (32.4%), or Group 2 Power Wheelchair (9.2%). The most common baseline AMD for the In-Person group were Cane/Crutches/Walker (24.1%), Non-Custom Manual Wheelchair (22.2%), and Custom Manual Wheelchair (15.7%).

The average FMA score before a wheelchair evaluation for the Telerehabilitation Group was 22.1 (SD 12.0), while the average score for the In-Person group was 29.5 (15.6). All FMA subcategories were significantly higher in the In-Person Group.

**Table 2:** Clinical Factors

Characteristic	Telerehabilitation Group (n = 1,669)	In-Person Group (n = 10,284)	p-value
Baseline Assistive Mobility Device			<.001
Cane/Crutch/Walker n (%)	481 (32.4%)	2,191 (24.1%)	
Group 1 Power Wheelchair n (%)	14 (0.9%)	62 (0.7%)	
Group 2 Power Wheelchair n (%)	149 (10.0%)	782 (8.6%)	
Group 3 Power Wheelchair n (%)	62 (4.2%)	1,217 (13.4%)	
Group 4 Power Wheelchair n (%)	1 (0.1%)	35 (0.4%)	
Group 5 Power Wheelchair n (%)	0 (0.0%)	11 (0.1%)	
Power Scooter n (%)	26 (1.8%)	259 (2.9%)	
Transport Wheelchair n (%)	39 (2.6%)	313 (3.4%)	
Custom Manual Wheelchair n (%)	50 (3.4%)	1,431 (15.7%)	
Non-Custom Manual Wheelchair n (%)	418 (28.2%)	2,017 (22.2%)	
No Device n (%)	244 (16.4%)	775 (8.5%)	
Functional Mobility Assessment (FMA) subcategories			
Daily Routine	2.1 (1.4)	3.1 (1.9)	<.001
Comfort Needs	2.0 (1.3)	2.7 (1.8)	<.001
Health Needs	2.2 (1.4)	2.9 (1.8)	<.001
Operating Independently/Safely/Efficiently	2.1 (1.4)	3.0 (1.9)	<.001
Reaching/Carrying Out Tasks Different Heights	2.2 (1.4)	2.8 (1.8)	<.001
Transfer from One Surface to Another	2.9 (1.6)	3.5 (1.8)	<.001
Personal Care	2.5 (1.5)	3.3 (1.9)	<.001
Indoor Mobility	2.4 (1.5)	3.3 (1.9)	<.001
Outdoor Mobility	1.9 (1.3)	2.7 (1.8)	<.001
Personal/Public Transportation	2.5 (1.6)	3.3 (2.0)	<.001
FMA overall mean (SD) (60 max score)	22.1 (12.0)	29.5 (15.6)	<.001

## Discussion

Individuals who are in need of AMD have limited access to therapists with seating and mobility expertise (Cooper et al., 1996; Hoenig et al., 2005; Bell et al., 2020). Telerehabilitation is an alternative modality not intended to completely replace traditional in-person services. This study accounted for health conditions and FMA scores for PwD which is consistent with previous publications (Cooper et al., 2002; Schein et al., 2010; Bell et al., 2020). Specifically, the results of this study indicate higher utilization of telerehabilitation for wheelchair evaluations in older adults aged 65 years and older, those with progressively acquired disabilities, and those with lower satisfaction measured by the baseline FMA Scores with their current means of mobility.

### Demographic Characteristics

Older adults, a generally more vulnerable population, may have sought telerehabilitation services at this time due to health and safety concerns during the COVID-19 pandemic. During the COVID-19 pandemic, adults over the age of 65 years or those with co-morbid conditions were encouraged to socially distance. This led to trends in lower utilization of in-person healthcare resources and increased use of telerehabilitation services (von Humboldt et al., 2022).

In the wake of the COVID-19 pandemic, telerehabilitation use surged because Medicare and private payers eased payment restrictions. Importantly, the Public Health Emergency changed the national landscape of payment and coverage equality and licensure. Beyond the Public Health Emergency declaration, the Centers for Medicare and Medicaid Services (CMS) acted to establish numerous flexibilities and waivers to provide delivery system stakeholders with the capability to adjust to best meet needs in the context of the COVID-19 pandemic (Centers for Medicare & Medicaid Services, n.d.).

There were several telerehabilitation-related actions during this time, indicating significant CMS activity to expand coverage and reimbursement for telerehabilitation during the pandemic (United States Department of Health and Human Services, 2020.). Specifically, the Consolidated Appropriations Act (CAA) 2023 establishes an extension of many key telerehabilitation policies allowed during the public health emergency through the end of 2024. Before the pandemic, telerehabilitation use was largely limited and restrained by the ambiguous and often changing regulations regarding reimbursement and licensure. Another significant challenge for the adoption of telerehabilitation is coverage equality. Like payment equality, there is a lack of consistency in how telerehabilitation services are to be covered by insurance plans. Some plans offer no coverage, other plans detail specific limitations on services, and others have full coverage. Due to the amount of funding sources available to individuals, each plan would need to be reviewed for specific telerehabilitation guidelines. It is difficult to determine why there is a discrepancy of specific payer types within the data, however, it is noted within the study limitations. For example, for most Medicaid benefits, federal Medicaid laws and regulations do not specifically address telerehabilitation delivery methods or the criteria for implementation of telerehabilitation. As a result, each state has broad flexibility in designing the parameters of telerehabilitation delivery methods. It is also noted that there was a significant difference in age between the two groups which could also have an impact on the types of insurance one would be allowed and/or purchase.

### Clinical Characteristics

Persons in the telerehabilitation group had lower FMA scores indicating a low satisfaction with mobility. People with lower satisfaction with their mobility may have difficulty accessing outpatient seating/mobility clinics and see telerehabilitation as a viable option for their wheelchair evaluation needs. The COVID-19 pandemic led everyone to make numerous changes in their lives during that time. Wheelchair users in need of a new wheelchair faced closed clinics and limited access to knowledgeable and experienced therapists who are essential to the wheelchair evaluation process. Having telerehabilitation services as an option for these individuals is beneficial still because according to a study that used the Functioning Everyday with a Wheelchair outcome tool (Mills et al., 2007), there were no significant differences in services for seating and mobility evaluations either in-person or via telerehabilitation, other than transportation considerations (Schein et al., 2010).

### Clinical Relevance

The use of telerehabilitation for wheelchair evaluations is a relatively new area of study. Previous publications have examined the increasing trend of telerehabilitation services (von Humboldt et al., 2022), the benefits of telerehabilitation services (Lemaire et al., 2001), and satisfaction with telerehabilitation services (Ott et al., 2020). However, a study has not yet analyzed the clinical and demographic characteristics of individuals utilizing telerehabilitation for new wheelchair evaluations. Descriptive research such as this study helps to provide initial insight into new concepts. Future causal/inferential studies will be more rigorous as the findings from this study will better inform hypotheses. In addition, future research studies should evaluate not only the effectiveness but also the cost-effectiveness of telerehabilitation compared to in-person care to influence reimbursement policy.

### Limitations

Several limitations deserve discussion. First, there were unequal sample sizes between the telerehabilitation and in-person groups. One group included users receiving in-person assessment compared to those within the registry who received a telerehabilitation assessment, which may have provided a different assessment experience for the PwD and ATP. However, the assessment protocol was standardized and used certified trained ATPs for consistency and best practice. An additional limitation of this study is that the data were collected during the COVID-19 pandemic so results may not be generalizable, however, the purpose of the paper was to describe patient characteristics of individuals receiving their wheelchair evaluation via telerehabilitation during this time. Additionally, this study is a retrospective cohort analysis, so causal inferences cannot be determined.

## Conclusion

This project is impactful as it will support larger comparative effectiveness studies contrasting the telerehabilitation to in-person AMD evaluations for different patient populations. Larger comparative effectiveness studies will also steer research, influence reimbursement policy, and facilitate translational research directly impacting occupational/physical therapy practice for AMD services via telerehabilitation. With the expansion of telerehabilitation services, best practices for wheelchair provision should be explored for different ages, diagnoses, and device types.
